# Capnocytophaga Meningitis: A Rare Case of Bacterial Infection With Neurological Manifestation

**DOI:** 10.7759/cureus.74927

**Published:** 2024-12-01

**Authors:** Hansen M Tang, Leanne Sowunmi, Eric Parnell, Benjamin S Avner

**Affiliations:** 1 Internal Medicine, Western Michigan University Homer Stryker M.D. School of Medicine, Kalamazoo, USA; 2 Microbiology, Bronson Healthcare, Kalamazoo, USA; 3 Infectious Disease, Western Michigan University Homer Stryker M.D. School of Medicine, Kalamazoo, USA

**Keywords:** capnocytophagia, gram-negative bacteremia, gram negative meningitis, new-onset seizure, pulmonary critical care

## Abstract

We discuss a case of meningitis caused by *Capnocytophaga canimorsus (C. canimorsus)* infection in a previously healthy elderly male who presented with altered mental status and new-onset seizures requiring intensive care. An open wound had served as an entry point for the infectious organism. After 25 days of treatment with meropenem, he recovered with persistent functional limitations and was discharged. We also engage in a review of the relevant literature and highlight the importance of identifying animal exposure in patients with meningitis.

## Introduction

Meningitis is defined as an inflammation of the fluids and tissues surrounding the brain and spinal cord, often due to infections. Infections from the bacteria *Capnocytophaga canimorsus (C. canimorsus)* in humans are a rare cause of meningitis [1]. This organism is found in the oral flora of canine and feline species. Infection caused by this organism is associated with a high rate of ICU admission and high mortality among patients who are elderly or immunocompromised [1-3]. *C. canimorsus* infections present with a broad range of symptomatic manifestations, ranging from mild flu-like symptoms to septic shock. It is a fastidious organism requiring a prolonged incubation period [3].

Patients with evidence of sepsis and bacterial infection with *Capnocytophaga* have poor clinical outcomes. Many of them develop hypotension or respiratory failure, requiring the use of vasopressors or intubation and ventilator management, leading to ICU admission. Per Hästbacka et al., while the rates of ICU admission and mortality vary, estimates have suggested that among patients who develop sepsis, 19-35% of those with *C. canimorsus* bacteremia will require ICU admission. Mortality among those admitted to the ICU with *Capnocytophaga* infection ranges from 19 to 22% [3]. While the United States lacks a comprehensive national surveillance system offering epidemiological data on *C. canimorsus*, a notable study conducted in the Netherlands and Denmark revealed an incidence rate of 0.67 cases per million per year and an incidence rate of 0.5 cases per million per year respectively [2]. 

Through this case report, we aim to provide a systemic overview of the diagnostic challenges, treatment strategies, and prognosis related to an elderly male with meningitis and a novel neurological manifestation. The high mortality rate and low incidence rate necessitate rapid identification of the risk factors associated with an infection with this organism. We believe that the neurological and abdominal findings we present in this report will help increase awareness and aid in the early diagnosis and identification of *C. canimorsus* meningitis, thereby leading to improved patient outcomes.

## Case presentation

An 84-year-old male with a past medical history of erythema nodosum on 5 mg prednisone daily, abdominal aortic aneurysm status post endovascular aneurysm repair, heart failure with reduced ejection fraction, and heart block with a pacemaker was brought in by emergency medical services after being found on the ground with lacerations on his left forehead and posterior scalp by his daughter. His last known contact with his family had been on the night before admission. His daughter reported that he had been running fevers for the past week, with no other possible inciting events other than a 3 mm laceration on his left hand six days ago. He reported taking an extra dose of prednisone 5 mg as he thought it worked as an antibiotic. Additional symptoms included forgetfulness of the events before collapsing on the ground, urinary incontinence, and confusion about his current whereabouts. He denied having a headache, cough, or abdominal pain. No sick contacts were reported. No tongue biting was reported before admission. The patient lived alone and had no pets or animals on his property.

On admission, his vital signs were as follows - temperature: 96.8 °F, systolic blood pressure: 128/66mmHg, respiratory rate: 22 breaths per minute, and heart rate: 80 beats per minute. On physical exam, he had left anterior and left temporal scalp lacerations that were actively bleeding, lacerations on his anterior left leg and base of his right thumb, as well as a bandaged laceration on his posterior left hand with edema and erythema. He was later found to be unresponsive, seen seizing with eyes deviated upward and right, with tonic movements of all four extremities. He was given 6 mg IV lorazepam due to concerns of status epilepticus. He was also given an infusion of propofol and levetiracetam, intubated, and admitted to the ICU for ventilator management. His lactic acid level was 6.7 mmol/L (normal range: 0.07-2.5), procalcitonin was 9.22 ng/mL (<0.5 ng/mL), and WBC was 18.8 10^9^/L (4.0-11.0).

A CT scan of the head without contrast was normal. CT angiography of the chest, abdomen, and pelvis raised suspicions of possible epiploic appendicitis. A lumbar puncture was performed due to a suspicion of meningitis. CSF analysis showed nucleated cells of 7336/uL (82% polymorphonuclear), and glucose of <2. He was initially treated with acyclovir, ampicillin, ceftriaxone, and vancomycin. During hospitalization, blood culture samples indicated gram-variable bacilli on both the aerobic and anaerobic blood agar plates after 33 hours of incubation at 95 °F with 5% CO₂, eventually confirmed as *C. canimorsus*. CSF culture results five days later revealed the same organism; no sensitives were reported by the lab on any positive culture due to a lack of standardized protocol. The result was confirmed by broad-based PCR testing (Karius) that showed *C. canimorsus* at 9084 DNA molecules per microliter. His antibiotic regimen was changed to meropenem two days after admission to cover for anaerobic bacteria from CT findings suggestive of appendicitis, and he continued to have a waxing and waning clinical course. Further questioning of his daughter revealed that a puppy had licked an open 3 mm wound on his left hand before the onset of symptoms.

**Figure 1:**
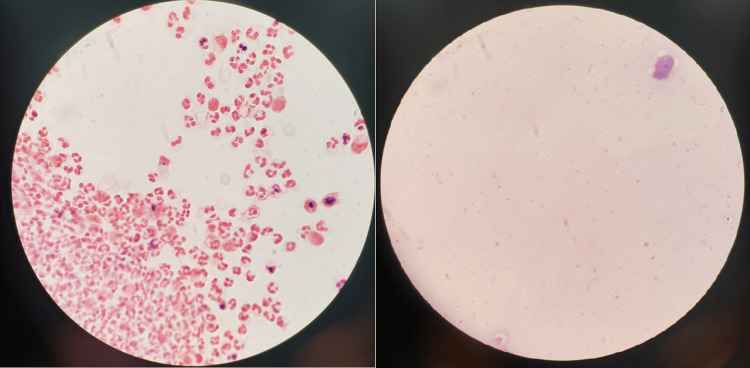
Capnocytophaga canimorsus identified via cerebral spinal fluid and blood cultures Left: specimen from cerebral spinal fluid with many polymorphonuclear cells and Capnocytophaga canimorsus. Right: Gram stain of blood culture revealing Capnocytophaga canimorsus

Table 1 presents the patient's CSF fluid analysis on admission. Figure 1 illustrates the detection of *C. canimorsus.*

**Table 1:** CSF fluid analysis on admission CSF: cerebral spinal fluid; RBC: red blood cells

CSF fluid parameter	Patient value	Reference range
Glucose CSF	2 mg/dL	50-75 mg/dL
Protein	897 mg/dL	15-45 mg/dL
Color	Yellow	Colorless
Character	Hazy	Clear
Volume	2.5 mL	
Nucleated cells	7336/uL	0-5/uL
RBC	1000/uL	
Polymorphonuclear %, CSF	82	
Mononuclear %, CSF	18	

The patient was febrile for the initial five days from admission with a maximum temperature of 102.8 °F. Continuous EEG monitoring showed no epileptiform features and slow nonreactive waveforms suggestive of moderate to severe encephalopathy. He was treated with 25 days of meropenem due to ongoing concerns for concurrent appendicitis and recurrent fever spells. Throughout his ICU stay, the patient was consistently obtunded and confused, giving incoherent responses. He was successfully extubated on the 16th day of admission with residual physical weakness and confusion requiring discharge to a skilled nursing facility. 

## Discussion

*C. canimorsus* is a bacterium in the family of *Flavobacteriaceae* and the genus *Capnocytophaga*. The genus *Capnocytophaga* comprises nine distinct species, primarily delineated by their habitation within the oral cavities of either humans or domesticated dogs and cats. Specifically, *C. cynodegmi*, *C. stomatis, C. canis, *and* C. canimorsus* colonize the oral cavity of dogs and cats, with *C. canimorsus* emerging as the predominant cause of infection from that genus stemming from dog bites [1-2]. *C. canimorsus* is a bacterial pathogen found primarily in the saliva of healthy dogs and cats and transmitted to humans primarily via dog bites [3-4]. It is a slow-growing, gram-negative, fermentative bacteria that is microaerophilic, capnophilic, exhibiting gliding motility. 

Capnocytophaga is a rare cause of meningitis. According to Butler et al., the average age at diagnosis of Capnocytophaga meningitis is 55 years, with males more commonly affected than females, at a rate of 3:1 [1]. The fatality rate is 26%, often manifesting in more severe presentations such as severe sepsis, septic shock, gangrene of digits, bacteremia, meningitis, endocarditis, neurological abnormalities, and eye infections. Bites or scratches from cats and dogs were suspected to be the primary sources of infection in 43% of cases [4]. Appendicitis in our patient was a suspected source of infection; however, repeat imaging showed its resolution with antibiotics, without overall improvement in mentation and clinical course. Risk factors for infection are highest in those with immunosuppression, splenectomy, and alcoholism. In a population-based study of rheumatoid arthritis patients, Wolfe et al. found an elevated risk of pneumonia requiring hospitalization with prednisone doses as low as 5 mg/day indicating chronic immunosuppression [5]. Our patient, who was on 5 mg/day of prednisone, was at a high risk for infection. 

*C. canimorsus* evades the immune response in the early stages by downregulating TLR4 and the proinflammatory cascade [6] and demonstrates resistance to phagocytosis, similar to *Neisseria meningitidis* *N. meningitidis*. In ICU cases, fatalities often result from severe sepsis [6]. It is unclear if the appendicitis in our patient was likely due to the alterations of the immune response by the organism. His obtundation and the subsequent development of status epilepticus upon admission are unique. A case series by Malik et al. [7] examining 31 cases of *C. canimorsus* meningitis revealed long-term hearing loss as the sole sequelae in 19% of the patients with no status epilepticus or residual mental decline on discharge. Despite the emphasis on the mortality associated with *C. canimorsus* in several case series [5-12], the morbidity, particularly in cases where patients present with status epilepticus necessitating intubation and subsequent residual mental decline, remains undocumented. 

The diagnosis of *C. canimorsus *is often missed or delayed, as 54% or about half of blood cultures and 70% of CSF are found to be positive after five days of incubation [8]. While incubation times are most commonly between two to seven days, in some cases, the organism can be detected in cultures up to 19 days later [9]. In CSF cultures that have remained negative, the use of PCR as well as MALDI-TOF mass spectrometry has also been often utilized from blood samples as a rapid alternative in providing early, accurate, and rapid diagnosis of slow-growing organisms such as *Capnocytophaga* [10]. Our medical facility did not have *Capnocytophaga* listed on our meningoencephalitis panel. We explored other options for diagnosis only after initial negative results and persistently worsening clinical course.

In our case, the CSF specimen was initially seen on the gram stain with many WBCs and many gram-negative bacilli. The morphology of the gram-negative bacilli displayed a pleomorphic spindle shape. This alone could not definitively identify the organism as *Capnocytophaga* species as other fastidious and anaerobic gram-negative bacilli could also have a similar appearance. It did help indicate that the organism was most likely not an enteric gram-negative bacillus and would most likely be a more rarely seen fastidious or anaerobic type of organism. The CSF specimen was plated to routine culture media [trypticase soy agar (TSA) with 5% sheep blood, Columbia CNA agar with 5% sheep blood, chocolate agar with hemoglobin, and MacConkey agar]. The TSA, CNA, and chocolate agar were incubated in 5% CO_2_ at 36 °C, and the MacConkey agar was incubated in ambient air at 36 °C. Due to the gram stain morphology, an additional TSA with 5% sheep blood was inoculated and incubated anaerobically to help recover any potential anaerobic organisms. In addition, Kairus PCR provided rapid confirmation of our diagnosis. 

Our patient exhibited several risk factors for *Capnocytophaga* bacteremia and meningitis, notably advanced age and chronic immunosuppression on prednisone 5 mg/day. Given the slow growth of the bacteria, a heightened degree of suspicion is imperative when managing patients who present with altered mental status and meningitis, particularly when there is a recent history of exposure to dogs and cats. While there is a higher incidence among patients with alcohol use disorder, immunosuppression, and asplenic conditions, these factors do not appear to be associated with disease severity [11]. 

*C. canimorsus* is generally susceptible to ampicillin, carbenicillin, carbapenems cephalothin, chloramphenicol, clindamycin, erythromycin, penicillin, and tetracycline and resistant to aminoglycosides and colistin and variably resistant to TMP-SMX [2]. Susceptibility was not reported from the lab with the reasoning being "There are currently no reproductive, definitive, or validated methods standards by which susceptibility testing results of this microorganism can be interpreted." No formal guidelines or consensus have been established for the treatment of *C. canimorsus* [12]. Antibiotic classes such as a third-generation cephalosporin have been considered; however, in our case, the severity of the illness including concern for hospital-acquired pneumonia six days after admission and the fluctuating clinical course while on other antibiotics ultimately led to treatment with a prolonged three-week course of meropenem, which was initiated after the detection of gram-negative rods in CSF fluid. This course was eventually continued for three weeks after diagnosis. In retrospect, utilization of a third-generation cephalosporin may have been more effective due to promising rates of recovery with the antibiotic class based on multiple case reports [7,12].

## Conclusions

This report highlights the importance of rapid identification of risk factors and that of considering *Capnocytophaga *in patients with meningitis and neurological symptoms without a bite wound. We described the progression of *Capnocytophaga* meningitis in an immunocompetent male who progressed into status epilepticus and required ICU care. The infectious disease team reached out to the Microbiology Department to continue blood cultures and for additional assistance in confirming the diagnosis of the pathogen. Challenges related to overreliance on PCR and the slow growth of *C. canimorsus* in traditional mediums led to a delay in prompt diagnosis. Sensitivities were not provided as no standardized protocol has been developed for this particular organism. Further research into the susceptibility of *C. canimorsus* will aid physicians in narrowing down antibiotic regimens and minimizing unnecessary side effects and financial burdens. Despite the absence of obvious bites or scratches, zoonoses should always be considered in severe cases of meningitis in elderly patients.
